# Soccer Attenuates the Asymmetry of Rectus Abdominis Muscle Observed in Non-Athletes

**DOI:** 10.1371/journal.pone.0019022

**Published:** 2011-04-25

**Authors:** Fernando Idoate, Jose A. L. Calbet, Mikel Izquierdo, Joaquin Sanchis-Moysi

**Affiliations:** 1 Radiology Department, Clínica San Miguel, Pamplona, Spain; 2 Department of Physical Education, University of Las Palmas de Gran Canaria, Palmas de Gran Canaria, Spain; 3 Department of Health Sciences, Public University of Navarre, Pamplona, Spain; Charité, Campus Benjamin Franklin, Germany

## Abstract

**Purpose:**

To determine the volume and degree of asymmetry of the *rectus abdominis* muscle (RA) in professional soccer players.

**Methods:**

The volume of the RA was determined using magnetic resonance imaging (MRI) in 15 professional male soccer players and 6 non-active male control subjects.

**Results:**

Soccer players had 26% greater RA volume than controls (P<0.05), due to hypertrophy of both the dominant (28% greater volume, P<0.05) and non-dominant (25% greater volume, P<0.01) sides, after adjusting for age, length of the RA muscle and body mass index (BMI) as covariates. Total volume of the dominant side was similar to the contralateral in soccer players (P = 0.42) and in controls (P = 0.75) (Dominant/non-dominant = 0.99, in both groups). Segmental analysis showed a progressive increase in the degree of side-to-side asymmetry from the first lumbar disc to the pubic symphysis in soccer players (r = 0.80, P<0.05) and in controls (r = 0.75, P<0.05). The slope of the relationship was lower in soccer players, although this trend was not statistically significant (P = 0.14).

**Conclusions:**

Professional soccer is associated with marked hypertrophy of the *rectus abdominis* muscle, which achieves a volume that is 26% greater than in non-active controls. Soccer induces the hypertrophy of the non-dominant side in proximal regions and the dominant side in regions closer to pubic symphysis, which attenuates the pattern of asymmetry of *rectus abdominis* observed in non-active population. It remains to be determined whether the hypertrophy of *rectus abdominis* in soccer players modifies the risk of injury.

## Introduction

Most soccer players have a favorite foot for kicking [1]. This preference induces muscle strength imbalances [2] and side-to-side differences in the cross-sectional area (CSA) of lower limb and trunk muscles [3]–[5]. The abdominal wall plays an important role in soccer. When kicking, abdominal muscles contribute to stabilize the body and are the main responsible of the powerful flexion and rotation of the trunk [6], [7]. Traditionally, muscle strains [8] and symphysis related injuries [9] have been associated to the asymmetric hypertrophy of abdominal muscles in professional soccer players. However, it remains to be determined whether soccer induces an asymmetric hypertrophy of abdominal muscles.

*Rectus abdominis* (RA) extends the length of the abdomen from the inferior costal margin to the pubic symphysis and is considered the main responsible of trunk flexion [10]. The recti are paired straplike muscles, separated at the midline by the *linea alba* [11]. In the non-active population both sides of RA have similar muscle volumes but follow different patterns of hypertrophy one from each other [12]. In the proximal regions, the dominant side (the corresponding to the side of the dominant arm) tend to be greater than the non-dominant side but RA changes to greater CSA and volumes in the non-dominant side in the more distal regions [12]. The practice of asymmetric sports can modify the pattern of hypertrophy of RA observed in the non-active population. In professional tennis players the non-dominant side is greater than the dominant side along the whole muscle, this asymmetry increases from the proximal to the distal region, from 18 to 55%, respectively [12]. In soccer, kicking demands repeated unilateral trunk flexion in the direction of the non-dominant side [7]. Whether soccer modifies the pattern and degree of hypertrophy of RA observed in the non-active population remains unknown.

The effect of soccer on RA muscle asymmetry is of clinical interest, especially in the region around the pubic symphysis. Chronic groin pain is a common pathology in soccer players that may be influenced by side-to-side differences into RA and *adductor longus* muscles [9]. These muscles are intimately associated because both attach to the anterior capsular soft tissues of the pubic symphysis [9]. Using MRI, Masuda et al. [4] found that adductor muscles of the non-dominant leg (analyzed together) were hypertrophied compared to the dominant leg. On the other hand, a recent study observed that soccer was not associated to a significant side-to-side asymmetry in the mean CSA of RA muscle [13]. However, the muscle volume of the whole RA was not determined in this muscle, and the most proximal and distal regions were not analyzed and compared to the pattern observed in non-active controls.

The hypothesis to be tested is that professional soccer is associated with an asymmetric development of the *rectus abdominis* muscle, with greater volume in the dominant compared to the non-dominant side in regions close to the pubic symphysis, reflecting greater stretch-shortening loads during kicking on the dominant *rectus abdominis*.

The main aim of this study was to determine the pattern and degree of hypertrophy of the musculus *rectus abdominis* (RA) in professional soccer players compared to non-active controls. A secondary aim was to determine if soccer induces an asymmetric hypertrophy of RA muscle in regions close to pubic symphysis.

## Methods

### Subjects

Fifteen male professional soccer players from a first division team of the Spanish football league and 6 non-active subjects (control group: CG) agreed to participate in the study and gave their written informed consent (Table 1). Participants of the CG had never been involved in regular physical exercise. All subjects were informed about the potential benefits and risks of the study and gave a written consent to participate. The study was approved by the ethical committee of the University of Las Palmas de Gran Canaria. All soccer players started soccer practice before 12 years old. In thirteen soccer players the dominant leg was the right leg, whilst 2 subjects had left leg dominance. In all subjects the side of the dominant leg was the same as the side of the dominant arm. In this article the dominant side of rectus abdominis muscle corresponds to the same side of the dominant leg, and vice versa.

**Table 1:** Physical characteristics of soccer players and control group and total and regional length of *rectus abdominis* from L1/L2 to pubic symphysis (mean ± SD).

Variables	Soccer	Controls
Age (years)	26.2±5.2	27.5±8.1
Height (cm)	182.3±5.6	177.7±2.6^a^
Body mass (Kg)	78.0±6.8	75.5±11.1
BMI	23.5±1.7	23.9±3.5
*Rectus abdominis* length (cm)		
1^st^ segment	3.1±0.3	3.7±0.5
2^nd^ segment	3.7±0.5	3.0±0.0^b^
3^rd^ segment	3.1±0.4	3.2±0.5
4^th^ segment	3.9±0.3	2.8±0.5^a^
5^th^ segment	3.0±0.0	3.3±0.5
6^th^ segment	3.9±0.3	3.0±0.0^b^
7^th^ segment	3.5±0.5	3.0±0.0^b^
8^th^ segment	3.9±0.3	2.8±0.4
Total	28.2±1.5	25.2±1.8^b^

a P<0.05 CG vs. SP.

b P<0.001 CG vs SP.

#### Magnetic resonance imaging

Magnetic resonance imaging was used to determine the muscle CSA and muscle volume of the left and right RA. A 1.5 T MRI scanner (Philips Achieva 1.5 Tesla system, Philips Healthcare, Best, the Netherlands) was used to acquire 10-mm axial contiguous slices from trunk, abdomen and pelvis, i.e., without interslice separation. Sagittal, coronal and transverse localizers of the body were obtained to determine precisely the anatomic sites for image acquisition. Transverse MRI images at rest (a breath-hold at mid expiration) oriented to be perpendicular to the anterior abdominal wall were obtained. Axial gradient-echo T1-weighted MR images was used with a repetition time of 132 ms and an echo time of 4.2 ms, flip-angle of 80° with a 42 cm^2^ field of view and a matrix of 256×256 pixels (in-plane spatial resolution 1.64 mm×1.64 mm). The body coil was used for image acquisition. The total research time was about 20 seconds which was within the breath-hold tolerance of all subjects.

The acquired MRI images were transferred to a computer for digital reconstruction to determine the CSA. The muscle volumes were calculated between L1-L2 discal level and the pubic symphysis. Each image was labeled referred to discal spaces, cranial aspect of coxofemoral joint and pubic symphysis using sagittal and axial scout images. All calculations were carried out by the same investigator, who was blinded to arm dominance, using a specially designed image analysis software (SliceOmatic 4.3, Tomovision Inc., Montreal, Canada), as described elsewhere [14]. A threshold was selected for adipose and lean tissues on the basis of the grey-level image pixel histograms to identify tissue area and the tissue boundaries were manually traced [14].

The total volume (V_total_) of the RA was assessed in each subject [15]. Regional RA volumes were also calculated for comparative purposes as described elsewhere [12]. The degree of side-to-side asymmetry was assessed by the calculation of a ratio of the volume of the dominant and non-dominant side [((non-dominant−dominant volume)×100))/dominant volume].

### Statistical analysis

Results are presented as means ± standard deviation, except for the bar figures, which represent means ± standard error of the mean. Side-to-side comparisons were carried out using the paired Student's t-test adjusted for multiple comparisons using the Bonferroni-Holm method. Analyses of covariance were performed to compare differences across groups, with age, BMI (body mass index) and total length of rectus abdominis muscle as covariates. Between-groups segment-to-segment comparisons were adjusted for the length of segment under scrutiny. The relationship between muscle length and muscle volumes into each group was determined by linear regression analysis. To test the similarity of slopes and intercepts of these relationships, the corresponding t-test was applied for the model: Y_ij_ = α_i_+β_i_X_ij_+ε_ij_ for i = 1,2 (1 = soccer players, 2 = controls) and j = 1,…, n_1_ being ε_ij_ i.i.d. random variables following a distribution N(0, σ_1_). SPSS package (SPSS Inc., Chicago, IL, USA, v15.0) for personal computers was used for the statistical analysis. Significant differences were assumed when P<0.05.

## Results

### Physical characteristics and length of rectus abdominis

Physical characteristics and total and regional length of *rectus abdominis* muscle are summarized in Table 1. Soccer players and controls were comparable in age, body mass and body mass index. Soccer players were significantly taller (P<0.05) and the length of the *rectus abdominis* was longer (P<0.001) than controls.

### Differences into each group

#### Muscle volumes

Table 2 summarizes total and regional muscle volumes in soccer players and controls. Total volume of the non-dominant side was similar to the dominant side in soccer players (P = 0.42) and in controls (P = 0.75). In soccer players and controls, the non-dominant segments 7 and 8 were hypertrophied compared to the dominant side (P<0.05), in the control group the dominant segment 2 was greater than the contralateral (P<0.05). Side-to-side differences were not statistically significant at the other segmental levels.

**Table 2:** Total and regional *rectus abdominis* muscle volumes (values expressed in cm^3^, mean ± SD) and asymmetries.

Segments	Soccer Players					Controls				
	Dominant	Non-dominant		Total	ASY(%)	Dominant	Non-dominant		Total	ASY(%)
**S1**	26.3±5.9	25.6±4.6	*P = 0.54*	51.9±9.9	0	20.0±3.3	20.8±5.4	*P = 0.57*	40.8±8.3	4
**S2**	32.0±8.1	31.2±7.6	*P = 0.62*	63.2±14.6	0	23.1±6.1	20.0±6.3	*P<0.05*	43.0±12.2	−14
**S3**	25.7±4.8	25.5±4.4	*P = 0.77*	51.3±8.8	0	20.6±4.3	18.8±2.7	*P = 0.49*	39.4±4.3	−4
**S4**	34.8±5.5	33.7±6.2	*P = 0.32*	68.5±10.9	−3	23.6±4.5	22.0±6.5	*P = 0.26*	45.6±10.7	−8
**S5**	26.7±3.9	28.0±4.4	*P = 0.12*	54.7±7.7	6	20.5±4.3	21.6±4.8	*P = 0.29*	42.1±8.8	5
**S6**	36.0±4.0	36.6±5.5	*P = 0.57*	72.7±8.8	2	21.9±3.1	23.4±4.3	*P = 0.17*	45.3±7.1	7
**S7**	28.7±6.6	30.9±7.0	*P<0.01*	59.6±13.3	8	19.0±2.4	20.7±2.0	*P<0.01*	39.7±4.3	10
**S8**	10.7±6.5	11.8±6.5	*P<0.01*	22.5±12.9	14	9.2±5.7	11.7±6.3	*P<0.01*	20.9±11.9	34
**Total**	220.9±26.8	223.4±25.3	*P = 0.42*	444.3±50.9	1	157.7±23.8	159.0±27.0	*P = 0.75*	316.7±67.7	1

Comparisons are made between dominant and non-dominant sides into each group.

**ASY:** Asymmetry between the dominant and non-dominant sides ((Non-dominant-Dominant)*100)/Dominant.

**Figure 1:**
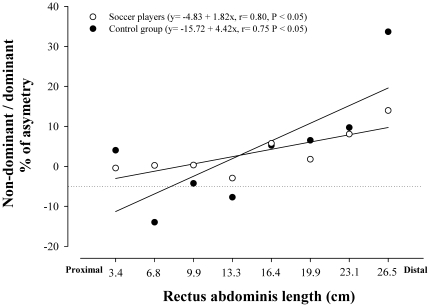
Relationship between the asymmetry in muscle volume of the dominant and non-dominant sides (expressed in percentage) and the *rectus abdominis* segments ordered in the rostro-caudal direction. In professional soccer players (white circles) and non-active subjects (black circles). Not significant differences were observed between the slopes and intercepts (P = 0.14 and P = 0.85, respectively).

A positive relationship was observed between muscle length starting from the inter-discal L1-L2 space and the degree of asymmetry in muscle volume expressed as the non-dominant/dominant ratio in soccer players (r = 0.80, P<0.05) and in controls (r = 0.75, P<0.05), being more asymmetric the more distal segments (Fig. 1). Not significant differences were observed between the slopes and intercepts when both groups were compared (P = 0.14 and P = 0.85, respectively).

#### Cross sectional area (CSA)

Table 3 summarizes the maximum CSA into each segment. In soccer players, similar side-to-side CSA was observed in all segments. In controls, segments 7 and 8 had a greater CSA in the non-dominant than in the dominant side (P<0.05), whilst no side-to-side differences were observed in segments 1 to 6. The maximum CSA of the non-dominant and dominant sides was positioned at a similar distance from the pubic symphysis in soccer players (12.0±8.1 vs 11.8±7.7 cm, respectively, P = 0.91) and in controls (15.9±7.6 vs 20.0±2.6 cm, respectively, P = 0.19).

**Table 3:** *Rectus abdominis* cross sectional areas (values expressed in cm^2^, mean ± SD) and asymmetries.

Segments	Soccer Players					Controls				
	Dominant	Non-dominant		Total	ASY(%)	Dominant	Non-dominant		Total	ASY(%)
**S1**	9.9±2.1	9.8±2.1	*P = 0.87*	19.7±3.9	1	8.6±0.7	8.5±1.7	*P = 0.84*	17.1±2.3	−2
**S2**	9.8±2.1	9.6±1.8	*P = 0.73*	19.3±3.6	1	8.2±1.9	7.6±1.8	*P = 0.11*	15.8±3.6	−7
**S3**	9.1±1.3	9.3±1.7	*P = 0.62*	18.4±2.8	3	7.6±1.5	7.0±1.2	*P = 0.46*	14.6±1.7	−5
**S4**	9.7±1.6	9.5±1.6	*P = 0.57*	19.2±2.9	−1	7.7±1.2	7.7±1.5	*P = 0.84*	15.4±2.7	0
**S5**	9.5±1.3	9.8±1.6	*P = 0.22*	19.3±2.7	4	7.5±1.1	8.0±1.4	*P = 0.33*	15.5±2.3	6
**S6**	9.7±1.1	9.8±1.2	*P = 0.84*	19.5±2.1	1	7.4±1.1	7.9±1.2	*P = 0.27*	15.2±2.2	7
**S7**	9.4±1.3	9.6±1.1	*P = 0.38*	19.0±2.2	4	7.1±1.2	7.7±1.0	*P<0.01*	14.7±2.1	8
**S8**	4.4±1.9	4.9±1.9	*P = 0.08*	9.3±3.6	16	4.0±2.0	5.0±1.8	*P<0.01*	9.0±3.8	33

Comparisons are made into each group between dominant and non-dominant sides.

**ASY:** Asymmetry between the dominant and non-dominant sides ((Non-dominant-Dominant)*100)/Dominant.

### Differences between groups

**Figure 2:**
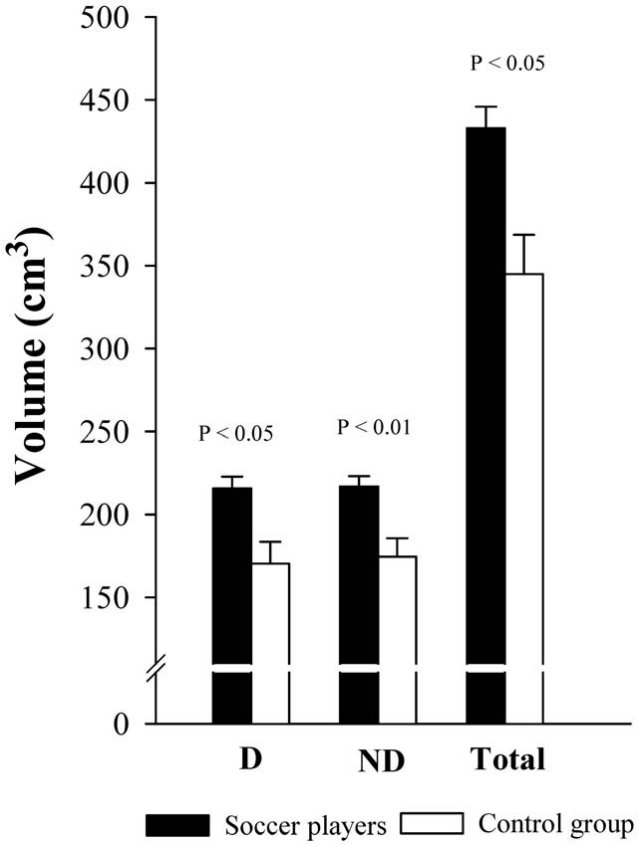
*Rectus abdominis* muscle volumes in professional soccer players and non-active subjects, after adjustment for the length of the *rectus abdominis*, age and BMI.

Muscle volume of RA was 40% greater in soccer players than in the control group (P<0.001). Compared to controls, soccer players had 40% (P<0.001) and 41% (P<0.001) more muscle volume in the dominant and non-dominant sides, respectively. After adjusting for age, the length of the RA muscle and BMI as covariates the total muscle volume of RA was 26% greater in soccer players than in the control group (P<0.05). Soccer players had also 28% (P<0.05) and 25% (P<0.01) more total muscle volume in the dominant and non-dominant sides compared to controls, respectively (Fig. 2).

**Figure 3:**
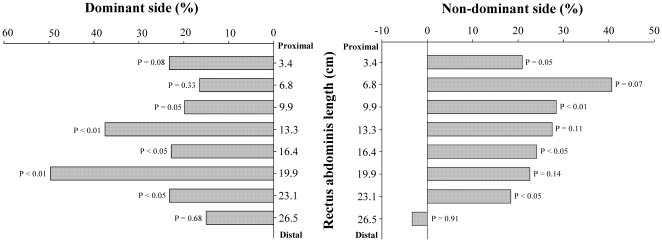
Differences between professional soccer players and non-active subjects in the muscle volume of the dominant and non-dominant *rectus abdominis* compared segment by segment, after adjustment for the length of the *rectus abdominis*, age and BMI.

**Figure 4:**
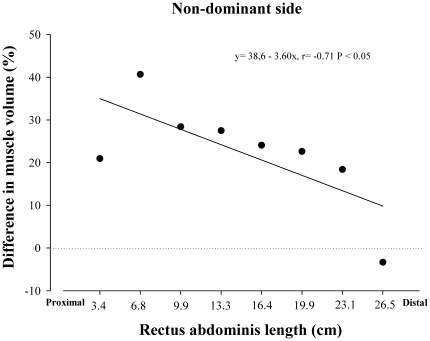
Relationship between muscle length starting from the inter-discal L1-L2 space and the mean difference in the muscle volume of the non-dominant *rectus abdominis*, in soccer players compared to controls, after adjustment for the length of the *rectus abdominis*, age and BMI.

Soccer players had higher muscle volumes than controls in the dominant and in the non-dominant sides in segments 1 to 7 (P<0.05), whilst between groups differences were not observed in segment 8 (P = 0.63 and P = 0.98, respectively). After controlling for age, the length of each segment and BMI as covariates, soccer players had higher muscle volumes than controls in segments 4–7 of the dominant side and 1, 3, 5 and 7 of the non-dominant side (Fig. 3). An inverse relationship was observed between muscle length starting from the inter-discal L1-L2 space and the mean difference in the muscle volume of the non-dominant side between soccer players and controls adjusted for age, the length of each segment and BMI (r = −0.71, P<0.05) (Fig. 4).

**Figure 5:**
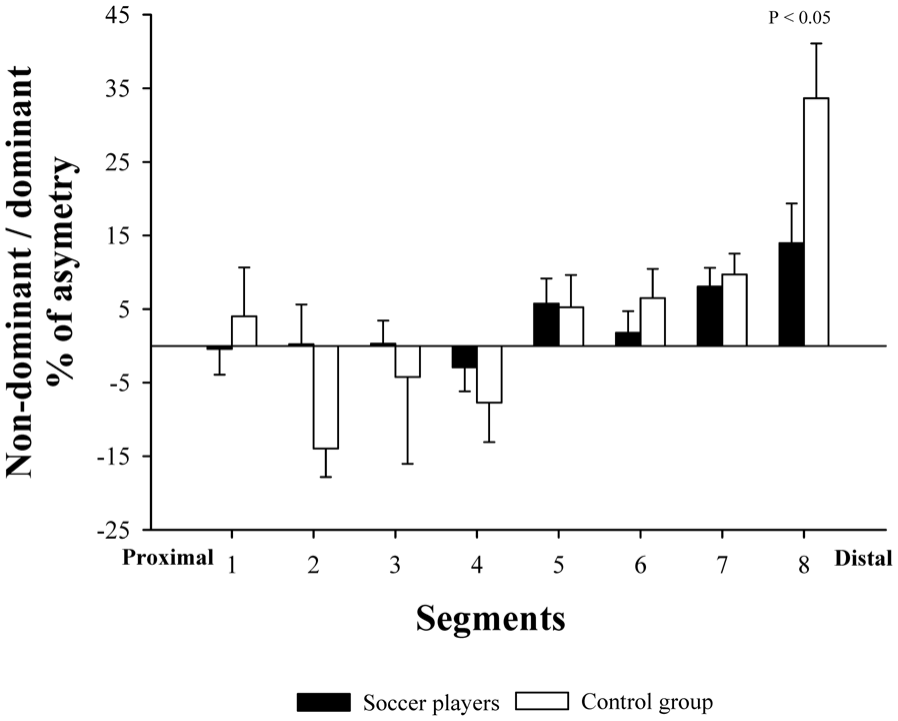
Differences between professional soccer players and non-active subjects in the percentage of asymmetry in the muscle volume of *rectus abdominis*, segment by segment.

The degree of side-to-side asymmetry was similar in soccer players than in controls (1.3±5.1 vs 0.7±6.1%, respectively, P = 0.82). Between group differences in the degree of asymmetry were also similar in all segments, except for segment 8, where controls were more asymmetric than soccer players (P<0.05)(Fig. 5).

## Discussion

In this study we have determined for the first time the volume and degree of asymmetry of the musculus *rectus abdominis* in professional male soccer players. Soccer was associated with 26% greater *rectus abdominis* volume (both sides considered together) than non-active subjects due to a similar increase of the muscle volume of the dominant and non-dominant sides (28 and 25%, respectively). Nevertheless, our study also shows that *rectus abdominis* is an asymmetric muscle in soccer players. The magnitude of asymmetry increased progressively from the proximal to the distal regions in soccer players and in non-athletes. This pattern of asymmetry was attenuated in the soccer players due to the hypertrophy of the non-dominant side in proximal regions and the dominant side in regions closer to pubic symphysis.

The present study shows that *rectus abdominis* muscle of professional soccer players adapts to the strength and power demands of this sport increasing a 26% its muscle volume. *Rectus abdominis* is submitted to very high loads playing soccer. When kicking fast stretch-shortening cycles of *rectus abdominis* allow trunk rotation and flexion to apply maximal power to the ball [7]. *Rectus abdominis* also contributes to stabilize the trunk and to maintain balance during many soccer actions, i.e. sudden starts and stops, rapid changes of directions or contacts with other players [16]–[18]. However, the degree of hypertrophy of RA in the soccer players can be considered low compared to other sports. A recent study observed that RA muscle of professional tennis players was hypertrophied a mean of 58% compared to non-active population [12]. However, the hypertrophy of RA was asymmetric, i.e. the non-dominant side was more hypertrophied than the dominant side (74 vs 29%, respectively) [12]. An interesting finding of the present study was that the magnitude of hypertrophy of RA in our soccer players (both sides together) was comparable to the hypertrophy described in the dominant side of the RA of professional tennis players (26 vs 29%, respectively) [12]. On the other hand, our soccer players had similar total CSA (both sides added) than elite wrestlers [19], [20] and judokas [19] (19, 21 and 19 cm^2^, soccer players from the present study, wrestlers and judokas, respectively), but our soccer players were significantly taller (+10 cm). These studies used images near the umbilicus to measure the CSA, which corresponds to distances between 3.9 and 14.3 cm above the pubic symphysis in the present study [19], [20]. Compared to young soccer players of similar height than wrestlers and judokas [19], [20], the total CSA of our professional players was slightly higher (age 17.6±0.5 yrs, total CSA 15 cm^2^; total CSA was calculated as the mean CSA from the mid level of L1/L2, L2/L3, L3/L4, L4/L5, and L5/S1) [21].

Our study also shows that soccer induced a similar increase in the muscle volume of both sides of RA (28 and 25%) which contributed to maintain the side-to-side proportions observed in non-active controls (ratio 0.99). This result is in concordance with a previous study conducted in professional soccer players who found that the mean CSA of both sides of RA was similar (mean CSA measured at three different segmental levels, midlevel of L2/L3, L3/L4 and L4/L5) [13]. However, despite the similar volumes in both sides of RA, the present study shows that RA of professional soccer players is an asymmetric muscle. As illustrated in figure 1, the magnitude of asymmetry increased progressively from the proximal to the distal region in both groups. Interestingly, the hypertrophy of the non-dominant side in the proximal regions and the dominant side in the regions closer to pubic symphysis observed in the soccer players contributed to attenuate the asymmetry of RA of non-active subjects. Although this trend was not statistically significant (P = 0.14) these results may suggest that a greater amount of exercise performed with the dominant leg could compensate for the asymmetric pattern of hypertrophy of RA induced by the predominant use of the dominant arm observed in control population.

The pattern of hypertrophy observed in soccer players is compatible with the asymmetric nature of kicking. When kicking abdominal muscles contribute to the maintenance of balance and stability [7], [17], [22]. Our study suggests that the non-dominant side hypertrophies to provide a solid foundation for the torques generated by the dominant leg [18]. The lineal decrease in the degree of hypertrophy of non-dominant RA from the distal to proximal segments observed in the soccer players is compatible with higher soccer-induced loading in the proximal segments of the non-dominant RA. On the other hand, the hypertrophy of the regions of the dominant RA closer to pubic symphysis (segments 4–7) could contribute to increase the force generating capacity and peak power of the dominant-leg [23], [24]. These results support the high strength and power demand of RA in soccer and suggest that the hypertrophy of RA muscle could play an important role when training to increase force production during kicking.

Common injuries in the lumbopelvic region could be associated to the pattern of hypertrophy of RA muscle observed in the soccer players, i.e., low back pain [25]–[27] or chronic groin pain [9]. Asymmetric sports using the upper extremities, i.e. tennis or cricket, increases the side-to-side asymmetry of abdominal muscles observed in non-athletes, which has been related to a higher risk of low back pain [12], [25], [28]. A recent study using electromyography has shown that as a mechanism of low back injury prevention [29], RA of soccer players activates earlier than in non-athletes in the presence of external perturbations [6]. It remains to be determined whether the pattern of hypertrophy of RA induced by soccer practice, turning this muscle more symmetric, could also contribute to reduce the risk of lower back pain [27]. On the other hand, the asymmetric hypertrophy of RA close to pubic symphysis has been associated to chronic groin pain in soccer players [9]. Our study shows that the increase in the muscle volume of the segments of the dominant RA closer to pubic symphysis (4–7) contributed to attenuate the asymmetry of RA muscle in the soccer players. However, the greatest contribution was made by the region closer to pubic symphysis (segment 8, mean 4 cm above pubic symphysis), which was the more pronounced along the longitudinal axis of RA (19%). Interestingly, in this region the RA of the soccer players was hypertrophied unilaterally, compared to controls the dominant RA had higher volumes but the non-dominant side was similar if not smaller. Future studies should analyze the influence of this pattern of hypertrophy of the dominant RA near pubic symphysis on the risk of chronic groin pain.

In summary, the present study describes for the first time the effects of professional soccer on the muscle volume of the *rectus abdominis*. Our study indicates that soccer is associated with 26% greater *rectus abdominis* volume (both sides considered together) due to a similar increase of the muscle volume of the dominant and non-dominant sides compared with a healthy control group. We have also shown that the *rectus abdominis* is asymmetric in soccer and in controls. The degree of asymmetry increases linearly from the inter-discal L1-L2 space to the pubic symphysis in soccer players and in non-athletes. This pattern of asymmetry is attenuated in the soccer players due to the hypertrophy of the non-dominant side in proximal regions and the dominant side in regions closer to pubic symphysis. It remains to be determined whether the pattern of hypertrophy of *rectus abdominis* modifies the risk of injury in soccer players. These results may be of great importance for coaches and clinicians to design more specific strength training and injury prevention programs.
